# A Unique GSK-3β inhibitor B10 Has a Direct Effect on Aβ, Targets Tau and Metal Dyshomeostasis, and Promotes Neuronal Neurite Outgrowth

**DOI:** 10.3390/cells9030649

**Published:** 2020-03-07

**Authors:** Xiao-Long Shi, Ning Yan, Ying-Jie Cui, Zhao-Peng Liu

**Affiliations:** Key Laboratory of Chemical Biology (Ministry of Education), Department of Medicinal Chemistry, School of Pharmaceutical Sciences, Shandong University, Jinan 250012, China; sxlmm0924@163.com (X.-L.S.); yandayanning@163.com (N.Y.); yingjiecui@sina.cn (Y.-J.C.)

**Keywords:** Alzheimer’s disease, Aβ, GSK-3β inhibitor, tau, metal dyshomeostasis, neurite outgrowth

## Abstract

Due to the complicated pathogenesis of Alzheimer’s disease (AD), the development of multitargeted agents to simultaneously interfere with multiple pathological processes of AD is a potential choice. Glycogen synthase kinase-3β (GSK-3β) plays a vital role in the AD pathological process. In this study, we discovered a novel 1*H*-pyrrolo[2,3-b]pyridine derivative B10 as a GSK-3β inhibitor that features with a quinolin-8-ol moiety to target the metal dyshomeostasis of AD. B10 potently inhibited GSK-3β with an IC_50_ of 66 ± 2.5 nM. At the concentration of 20 μM, B10 increased β-catenin abundance (β-catenin/GAPDH: 0.83 ± 0.086 vs. 0.30 ± 0.016), phosphorylated GSK-3β at Ser9 (p-GSK-3β/GAPDH: 0.53 ± 0.045 vs. 0.35 ± 0.012), and decreased the phosphorylated tau level (p-tau/GAPDH: 0.33 ± 0.065 vs. 0.83 ± 0.061) in SH-SY5Y cells. Unlike other GSK-3β inhibitors, B10 had a direct effect on Aβ by inhibiting Aβ_1-42_ aggregation and promoting the Aβ_1-42_ aggregate disassociation. It selectively chelated with Cu^2+^, Zn^2+^, Fe^3+,^ and Al^3+^, and targeted AD metal dyshomeostasis. Moreover, B10 effectively increased the mRNA expression of the recognized neurogenesis markers, GAP43, N-myc, and MAP-2, and promoted the differentiated neuronal neurite outgrowth, possibly through the GSK-3β and β-catenin signal pathways. Therefore, B10 is a potent and unique GSK-3β inhibitor that has a direct on Aβ and serves as a multifunctional anti-AD agent for further investigations.

## 1. Introduction

Alzheimer’s disease (AD) is a progressive neurodegenerative disease and the most common dementia in old-age people [1]. Histopathologically, the hallmarks of AD include the neuronal loss, the intracellular neurofibrillary tangles (NFTs), and the extracellular senile plaques formed mainly by the amyloid β-protein (Aβ) aggregation [2,3]. At present, the FDA-approved anti-AD drugs are cholinesterase inhibitors, donepezil, rivastigmine, tacrine, galantamine, and NMDA receptor antagonist memantine [4,5]. These drugs provide only limited efficiency and curable drugs remain lacking. Several hypotheses are put forth to explain the causes of AD, mainly including the amyloid cascade hypothesis [6,7], tau pathology [8,9], cholinergic hypothesis [10,11], inflammatory [12,13], metal ion dyshomeostasis [14,15,16,17], and oxidative stress [18,19]; however, the exact AD etiology is still unclear. Since AD is a complicated disease with multifaceted pathogenesis, the one-drug–one-target strategy is not very effective, and therefore, the multitarget-directed ligands that simultaneously interfere with the multiple processes of AD progression are more hopeful [20,21,22,23].

Glycogen synthase kinase-3 (GSK-3), a serine/threonine kinase largely expressed in the central nervous system (CNS), plays important roles in metabolism, proliferation, and apoptosis [24]. The two subtypes, GSK-3α (51 kDa) and GSK-3β (47 kDa), are 98% identical in their respective catalytic domains [25]. GSK-3β is predominantly the main isoform in most brain areas and the key kinase in AD responsible for the abnormal hyperphosphorylation of the microtubule-associated tau protein [26,27,28]. The hyperphosphorylated tau impairs the interaction between tau proteins and microtubules, leading to the detachment of tau from microtubules, destabilizing the microtubules in the neurons. The accumulation of hyperphosphorylated tau further generates paired helical filaments and subsequent aggregates to form the intracellular NFTs, one important biomarker of AD [29,30,31]. Moreover, increased GSK-3β activity may induce Aβ formation through its regulation of γ-secretase in the cleavage of the amyloid-precursor protein (APP) [32]. Overactivation of GSK-3β is also involved in neuroinflammation, neuronal death, and apoptosis through cell signal pathways [33,34]. Both in AD model and preclinical and clinical studies, GSK-3β has been proven as a therapeutic target for AD [35,36,37,38].

In an effort to find potent GSK-3β inhibitors to target multifacets of AD [39], we report here a unique and potent GSK-3β inhibitor, 6-(5-(4-((pyridin-4-ylamino)methyl)phenyl)-1*H*-pyrrolo[2,3-*b*]pyridin-3-yl)quinolin-8-ol (B10), which has a direct effect on Aβ targets tau and metal dyshomeostasis, and promotes neuronal neurite outgrowth.

## 2. Materials and Methods

### 2.1. Chemicals, Reagents, Cell Lines, and Antibodies

All the chemicals and reagents for the synthesis of B10 were commercially available or prepared according to the cited literature. A Bruker-600 NMR spectrometer (Brucker Co., Ltd., Zurich, Switzerland) was used to measure the ^1^H and ^13^C NMR spectra. High-resolution mass spectra (HRMS) were determined on a 6520 QTOF instrument (Agilent Technologies Inc., Santa Clara, CA, USA) by electrospray ionization (ESI). Melting points were measured on an X-6 micromelting point apparatus (Beijing Tech. Co., Ltd., Beijing, China). Column chromatography was performed on silica gel (200–300 mesh). In cell assay, compound B10 was dissolved in 100% dimethylsulfoxide (DMSO, Sigma, Shanghai, China) to obtain a 2 mM stock solution, the stock solutions were diluted in culture medium at various concentrations before each treatment, and the final DMSO concentration did not exceed 0.05% (*v*/*v*). SH-SY5Y cell line was provided by JiangShu KeyGen Biotech Co., Ltd. GSK3β was purchased from CarnaBio (Natick, MA, USA). Aβ_1-42_ (ChinaPeptides Co., Ltd., Shanghai, China) was dissolved in 100% DMSO to form a 2 mM stock solution immediately before use. p-GSK3β (Ser9) antibody was obtained from Abcam China (Shanghai). The β-catenin and p-tau (Ser396) were purchased from JiangShu KeyGen Biotech Co., Ltd. All other chemicals or reagents were of analytical grade and were obtained from Sigma Chemical Co. (Shanghai, China).

### 2.2. The Synthesis of 6-(5-(4-((Pyridin-4-ylamino)methyl)phenyl)-1H-Pyrrolo[2,3-b]pyridin-3-yl)quinolin-8-ol (B10)

To a solution of 5-bromo-3-iodo-1-(phenylsulfonyl)-1*H*-pyrrolo[2,3-*b*]pyridine (1, 5.56 g, 12 mmol) and 8-methoxy-6-(4,4,5,5-tetramethyl-1,3,2-dioxaborolan-2-yl)quinoline (4.11 g, 14.4 mmol) in dioxane (16 mL) and distilled water (0.8 mL) was added Pd(dppf)Cl_2_ (0.35 g, 0.48 mmol) and K_2_CO_3_ (4.38 g, 36 mmol). The mixture was stirred and refluxed for 2.5 h. Distilled water (10 mL) was added, and the resulting solution was extracted with dichloromethane. The organic phase was washed with brine and dried over anhydrous Na_2_SO_4_. After filtration and evaporation, the resulting solid was dissolved in methanol (60 mL), and a 6 mol/L of potassium hydroxide aqueous solution (20 mL) was added. After stirring at 60 °C for 1.5 h, the mixture was cooled to room temperature and neutralized with saturated potassium hydrogen sulfate aqueous solution. The mixture was extracted with AcOEt, washed with brine, and dried over anhydrous Na_2_SO_4_. After filtration and evaporation, the resulting yellowish solid was dissolved in 40% hydrobromic acid and refluxed for 24 h. The mixture was cooled to room temperature and neutralized with potassium hydroxy aqueous solution. The mixture was extracted with AcOEt, washed with brine, and dried over anhydrous Na_2_SO_4_. After filtration and evaporation, the residue was purified by silica gel chromatography (MeOH/CH_2_Cl_2_ = 1:30) to give 2 (1.3 g, 47.8%) as a red solid, m.p. > 240 °C; ^1^H NMR (400 MHz, DMSO-d_6_) δ 12.30 (s, 1H), 9.90 (s, 1H), 8.79 (d, *J* = 2.7 Hz, 1H), 8.67 (d, *J* = 1.9 Hz, 1H), 8.45 (d, *J* = 8.3 Hz, 1H), 8.38 (d, *J* = 1.9 Hz, 1H), 8.15 (s, 1H), 7.80 (s, 1H), 7.56 (dd, *J =* 8.3, 4.1 Hz, 1H), 7.50 (d, *J* = 1.4 Hz, 1H); ^13^C NMR (150 MHz, DMSO-d_6_) δ 154.03, 148.05, 147.83, 143.59, 138.07, 136.48, 133.89, 130.16, 129.93, 127.22, 122.58, 119.49, 114.48, 114.23, 112.11, 110.68; HRMS (ESI): Calcd. for C_16_H_10_BrN_3_O [M+H]^+^: 340.0080, found: 340.0086.

To a solution of 2 (910 mg, 2.68 mmol) in DMF (6 mL) was added imidazole (806 mg, 5.35 mmol) and TBSCl (364 mg, 5.35 mmol). The mixture was stirred at room temperature for 30 min. Distilled water (10 mL) was added, and the resulting mixture was extracted with AcOEt. The organic phase was washed with brine and dried over anhydrous Na_2_SO_4_. After filtration and evaporation, the residue was purified by silica gel chromatography (Hexane/AcOEt = 5:1) to give 3 (722 mg, 60%) as a white solid, m.p. 232–233 °C; ^1^H NMR (600 MHz, DMSO-d_6_) δ 12.32 (s, 1H), 8.82–8.78 (m, 1H), 8.64 (d, *J* = 2.1 Hz, 1H), 8.47–8.43 (m, 1H), 8.38 (d, *J* = 2.1 Hz, 1H), 8.18 (s, 1H), 7.94 (d, *J* = 1.7 Hz, 1H), 7.54–7.50 (m, 2H), 1.06 (s, 9H), 0.27 (s, 6H); ^13^C NMR (150 MHz, DMSO-d_6_) δ 152.77, 148.46, 148.08, 143.63, 140.62, 136.40, 133.43, 130.47, 130.08, 127.36, 122.39, 119.47, 117.29, 117.01, 113.83, 112.12, 26.33, 19.09, -3.39; HRMS (ESI): Calcd. for C_22_H_24_BrN_3_OSi [M+H]^+^: 454.0945, found: 454.0953.

To a solution of 3 (150 mg, 0.33 mmol) and *N*-(4-(4,4,5,5-tetramethyl-1,3,2-dioxaborolan-2-yl)benzyl)pyridin-4-amine (205 mg, 0.66 mmol) in toluene (3 mL), EtOH (1.0 mL) and distilled water (2.0 mL) was added Pd(PPh_3_)_4_ (27 mg, 0.023 mmol) and Na_2_CO_3_ (106 mg, 1.0 mmol). The mixture was stirred at 100 °C for 6 h. Distilled water (5 mL) was added, and the resulting solution was extracted with dichloromethane. The organic phase was washed with brine and dried over anhydrous Na_2_SO_4_. After filtration and evaporation, the resulting yellowish solid was dissolved in THF (5 mL), and TBAF (173 mg, 0.66 mmol) was added. After the mixture was stirred at room temperature for 30 min, distilled water (5 mL) was added. The resulting mixture was extracted with AcOEt, and the organic phase was washed with brine and dried over anhydrous Na_2_SO_4_. After filtration and evaporation, the residue was purified by silica gel chromatography (AcOEt/MeOH = 5:1) to give B10 (47 mg, 32%) as a yellow solid, m.p. >240 °C; ^1^H NMR (400 MHz, DMSO) δ 12.13 (d, *J* = 2.2 Hz, 1H), 8.78 (dd, *J* = 4.1, 1.6 Hz, 1H), 8.59 (s, 2H), 8.43 (dd, *J* = 8.4, 1.5 Hz, 1H), 8.32 (t, *J* = 5.6 Hz, 1H), 8.11 (d, *J* = 6.8 Hz, 2H), 8.08 (d, *J* = 2.6 Hz, 1H), 7.84 (d, *J* = 1.6 Hz, 1H), 7.81 (d, *J* = 8.2 Hz, 2H), 7.58–7.52 (m, 2H), 7.49 (d, *J* = 8.2 Hz, 2H), 6.78 (d, *J* = 6.8 Hz, 2H), 4.52 (d, *J* = 5.6 Hz, 2H); ^13^C NMR (100 MHz, DMSO) δ 171.39, 155.36, 152.92, 148.17, 146.64, 143.55, 141.50, 137.43, 136.92, 136.16, 135.35, 133.50, 128.86, 128.10, 127.42, 126.81, 125.14, 125.03, 121.44, 116.74, 113.73, 113.44, 109.84, 44.43; HRMS (ESI): Calcd. for C_28_H_21_N_5_O [M+H]^+^: 444.1819, found: 444.1835.

### 2.3. Glycogen Synthase Kinase-3β (GSK-3β) Kinase Assay

The inhibitory activity of B10 against GSK-3β was determined by the caliper mobility shift assay and followed the manufacturer protocol, using staurosporine as a positive control. Staurosporine or B10 was tested from 1 µM or 5 µM, 3-fold dilution, in IC_50_ determination. The kinase reaction was done in 96-well plate (Corning, Los Altos, MA, USA). Each well was loaded with compound and GSK-3β. The mixture was incubated at room temperature for 10 min. The reaction was started by the addition of peptide FAM-P15 (GL Biochem, Shanghai, China) and ATP (Sigma, Shanghai, China) prepared in reaction buffer. After incubation at 28 °C for 1 h, a stop buffer (25 μL) was added. The stopped reaction was analyzed on a LabChip EZ Reader (PerkinElmer, Shanghai, China) to give the conversion data at each concentration through the direct detection of both substrate and product via Laser-Induced Fluorescence (LIF) at 492 nm. The IC_50_ values were then calculated from dose-response curves using XLfit (curve fitting software for Excel).

### 2.4. Cell Viability Assay

SH-SY5Y human neuroblastoma cells (ATCC, CRL-2266^™^) were cultivated in Dulbecco’s modified Eagle’s medium DMEM/F-12 containing of 10% FBS, 1% penicillin, and 1% streptomycin and seeded in 96-well plates (Corning, Los Altos, MA, USA) at a density of 1 × 10^5^ in 100 μL medium per well, respectively, and kept in 5% CO_2_ atmosphere at 37 °C for 24 h. Compound B10 in different concentrations (3.125 μM, 6.25 μM, 12.5 μM, and 25 μM) in 100 μL medium were added, and the mixture was incubated another 24 h. Then, 20 μL of 3-(4,5-dimethyl-2-thiazolyl)-2,5-diphenyl-2H-tetrazolium bromide (MTT, 2.5 mg/mL) was added, and the cells were incubated at 37 °C for another 4 h. After the addition of 200 μL DMSO to dissolve the formazan crystals, the absorbance at the wavelength of 570 nm was measured with a SpectraMax M5 multimode plate reader (Molecular Devices, Shanghai, China). The data were analyzed by GraphPad Prism 5 software (GraphPad Software Inc., San Diego, CA, USA).

### 2.5. Western Blot Analysis on β-catenin, GSK-3β, and Tau Phosphorylation

SH-SY5Y cells were cultured in 90% DMEM/F12 and 10% PBS at a humidified atmosphere with 5% CO_2_. 1 × 10^6^ cells were seeded in 12-well plates (Corning, Los Altos, MA, USA) and incubated with compound at the indicated concentrations for 1 h and Aβ_25-35_ (Sigma, Shanghai, China) for another 6 h. After the SH-SY5Y cells were lysed with RIPA buffer, total protein concentration was determined by Bradford method. Cellular protein was mixed with an equal volume of SDS (sodium dodecyl sulfate) loading buffer (JiangShu KeyGen Biotech, Nanjing, China) and separated by electrophoresis (Bio-rad Power Supplies Basic, Shanghai, China) in polyacrylamide gel. Proteins were transferred from acrylamide gels to nitrocellulose membranes (Amersham Italia, Milan, Italy) and blocked in a blocking buffer (PBS, 5% nonfat milk) for 1.5 to 2 h at 20 °C. After overnight incubation at 4 °C with antibody p-GSK3β-Ser 9 (KaiTai-Bio, Hongzhou, China), or p-tau Ser396 (Abcam, Shanghai, China), or β-catenin antibody (Cell Signaling Technology, Danvers, MA, USA), and GAPDH (Santa Cruz Biotechnology, Shanghai, China), the blots were washed in Tween 20-TBS (TBST, JiangShu KeyGen Biotech, Nanjing, China) for 20 min and then incubated with secondary antibody (IgG-HRP; JiangShu KeyGen Biotech, Nanjing, China) for 1 h at room temperature. The blots were washed by TBST for 20 min and incubated with ECL chemiluminescent reagent (JiangShu KeyGen Biotech, Nanjing, China) for 3 min. Quantification of pixel intensity was done using gel imaging system (SYNGENE G:BOX/iChemi XR5, ISS, San Diego, CA, US) and Gel-Pro32 software (Media Cybernetics, Bethesda, MD, USA). GAPDH was used as an internal control.

### 2.6. Metal Chelation

Compound B10 was dissolved in 100% methanol to form a 20 µM solution. The salt (NaCl, KCl, CaCl_2_, MgCl_2_, FeSO_4_, ZnCl_2_, CuCl_2_, and AlCl_3_) was dissolved in 100% methanol to give a 40 µM metal ion solution. A mixture of the B10 solution (20 µM, 2 mL) with the metal ion solution (40 µM, 2 mL) or with 100% methanol (2 mL) was incubated at 25 °C for 30 min. The UV spectra of B10 alone or in the presence of each metal ion were recorded by TU-1901 ultraviolet–visible spectrophotometer (Beijing PurSee General Co, Ltd, Beijing, China) at the wavelength ranging from 200 to 500 nm, respectively.

### 2.7. Amyloid β-Peptide 1-42 (Aβ_1−42_) Aggregation and Cu^2+^-Induced Aβ_1−42_ Aggregation Assay

The 2 mM Aβ_1−42_ stock solution (10 μL) was diluted with 20 µM HEPES (pH = 6.6, 150 µM NaCl, 490 μL) into a 40 µM solution. Compound B10 and the positive control curcumin (Sigma, Shanghai, China) were dissolved in 100% DMSO to form a 2 mM stock solution. The stock solution (10 μL) of B10 or curcumin was diluted with 20 µM HEPES (490 μL) into a 40 µM solution. CuCl_2_ was dissolved in HEPES to give a 40 µM solution. Thioflavin T (ThT; Sigma, Shanghai, China) was dissolved in 50 mM glycine–NaOH buffer (pH = 8.0) to form a 5 μM stock solution. Aβ_1−42_ (20 μL, 40 µM), Aβ_1−42_ (20 μL, 40 µM) plus CuCl_2_ (20 μL, 40 µM), and Aβ_1−42_ (20 μL, 40 µM), and CuCl_2_ (20 μL, 40 µM) in the presence of compound (40 μL, 40 µM), were incubated at 37 °C for 24 h. After that, a 5 μM ThT stock solution was added to each well with a final volume of 200 µL/well and further incubated for 5 min. The fluorescence was measured on a Tecan Infinite 200 PRO (Tecan Trading Co., Ltd., Shanghai, China) at excitation and emission wavelengths of 450 nm and 490 nm, respectively. The inhibition rate is calculated based on the fluorescence differences by the formula: [1-(FL_compound_–FL_blank_)/FL_control_] × 100, both for the aggregation and disaggregation assay. The HEPES solution containing only compound was used as a blank group; the Aβ_1−42_ or Aβ_1−42_ /CuCl_2_ was the control group.

### 2.8. Disaggregation of Aβ_1−42_ Aggregation and Cu^2+^-Induced Aβ_1−42_ Aggregation

The positive control clioquinol (CQ, Santa Cruz Biotechnology, Shanghai, China) was dissolved in 100% DMSO to form a 2 mM stock solution. The stock solution of CQ (10 μL) was diluted with 20 µM HEPES (pH = 6.6, 150 µM NaCl, 490 μL) into a 40 µM solution. Aβ_1−42_ (20 μL, 40 µM) or Aβ_1−42_ (20 μL, 40 µM) plus CuCl_2_ (20 μL, 40 µM) was incubated in black, opaque 96-well plates (Corning, Los Altos, MA, USA) at 37 °C for 24 h to form Aβ_1−42_ aggregates. After that, 40 μL HEPES or B10 (40 μL, 40 µM) or CQ (40 μL, 40 µM) was added and incubated for another 24 h. A 5 μM ThT stock solution was added to each well with a final volume of 200 µL/well. After incubation for 5 min, the fluorescence was measured on a Tecan Infinite 200 PRO (Tecan Trading Co., Ltd., Shanghai, China) at excitation and emission wavelengths of 450 nm and 490 nm, respectively.

Aggregation and disaggregation of Aβ_1−42_ during the ThT assays were confirmed by transmission electron microscopy (TEM). Briefly, aliquots (10 μL) of the samples were taken from the wells and placed on a carbon-coated copper grid for 20 min. Each grid was stained with aqueous phosphotungstic acid (2%, 10 μL) for 1 min. After draining the excess staining solution, the specimen was transferred for imaging by TEM (Hitachi HT7700, Tokyo, Japan).

### 2.9. Neuronal Neurite Outgrowth Assay and Quantitative Real-Time Reverse Transcription-PCR (RT-PCR)

SH-SY5Y cells were cultivated in DMEM containing 10% FBS, 100 IU/L penicillin, and 10 μg/mL streptomycin and seeded in 96-well plates (Corning, Los Altos, MA, USA) at a density of 5 × 10^3^ in 100 μL medium per well and kept in 5% CO_2_ atmosphere at 37 °C for 24 h. After that, compound (RA or B10) diluted in medium (100 μL) was added and cultivated for 72 h. The morphology of neurite outgrowth was examined under an inverted microscope (2 × 100; Olympus, Tokyo, Japan).

After the SH-SY5Y cells were cultivated for 24 h, total RNA was extracted using TRIzol reagent (Thermo Fisher Scientific Inc., Carlsbad, CA, USA) according to the manufacturer directions. RNA was quantified by measuring the absorbance at 260 nm, and purity was determined by valuating the absorbance 260/280 nm ratio. The absorbance was measured by Shimadzu UV-2450 (Shimadzu China Co. Ltd., Shanghai, China). PrimeScript RT Master Mix (Takara Bio Inc., Shiga, Japan) was used to synthesize the first-strand cDNA. The determination of mRNA levels was determined by using SYBR Green Master Mix (Takara Bio Inc., Shiga, Japan). Primer sequences were as follows: GAP43, GAGCAGTTCGACCTAGTCCTT (upstream), GGTTGCGGCCTTATGAGCTT (downstream); N-myc, AGTTTGACTCGCTACAGCCC (upstream), GCAGCAGCTCAAACTTCTTCC (downstream); MAP2, TCTGCACACTCACATCCACC (upstream), CTGTGACCCATGCTCTCCAA (downstream); GAPDH, CAAATTCCATGGCACCGTCA (upstream), AGCATCGCCCCACTTGATTT (downstream). The GAPDH mRNA was used as the reference.

### 2.10. Molecular Docking

The protein structures of GSK-3β (PDB ID: 5F95) and Aβ_1−42_ monomer (PDB codes: 1IYT) were obtained from the RCSB Protein Date Bank. The protein structure was prepared with the SYBYL-X suite (version 2.1.1, Tripos). For GSK-3β, the ligand was first extracted from the protein, and then the hydrogen atoms and charges were added. The extracted ligand was used as a standard to generate the protomol. For Aβ_1−42_, hydrogen atoms were added to the crystal and charges were added to biopolymer by AMBER7 FF99 force field. The protomol was generated according to residues 13–27 (HHQKLVFFAEDVGSN). The 3D structures of B10 were prepared by the Sketch module of Sybyl followed by energy minimization using the Tripos force field. The Surflex-dock module was used for the simulations, and the related parameters implied in the program were kept at default.

### 2.11. Statistics

Qualitative data including the immunoblots and images are representatives of at least three independent experiments and expressed as means ± SD. Statistical differences between two groups were determined by the two-tailed unpaired or paired Student t-test. *p* < 0.05 is considered significantly different.

## 3. Results

### 3.1. Design and Synthesis of B10

**Figure 1:**
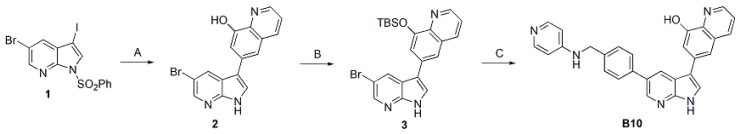
Synthesis of B10. Reagents and conditions: (A) (1) 8-methoxy-6-(4,4,5,5-tetramethyl-1,3,2-dioxaborolan-2-yl)quinoline, Pd(dppf)Cl_2_, K_2_CO_3_, dioxane/H_2_O, reflux, 2.5 h; (2) KOH, MeOH, 60 °C, 1.5 h; (3) 40% HBr, reflux, 24 h; (B) TBSCl, imidazole, DMF, 0.5 h; (C) (1) *N*-(4-(4,4,5,5-tetramethyl-1,3,2-dioxaborolan-2-yl)benzyl)pyridin-4-amine, Pd(PPh_3_)_4_, Na_2_CO_3_, toluene, EtOH, H_2_O, 100 °C, 6 h; (2) TBAF, THF, 0.5 h.

Metal dyshomeostasis plays important roles in AD pathogenesis by preceding or inducing NFTs, senile plaques, and reactive oxygen species (ROS) [14,15,16,17]. Cu^2+^, Zn^2+^, and Fe^2+^ are known to excessively exist in the senile plaques [17,40]. As one of the main cationic elements in plaque formation, copper ion binds to Aβ, promoting its accumulation and inducing ROS generation and oxidative stress [41,42]. The generated ROS may in turn produce modified Aβ species that favor Aβ aggregation and resist disaggregation [43,44]. 8-Hydroxyquinoline is a bidentate metal chelator and one of the most-used metal-chelating scaffolds in the design of multitarget AD agents [41,45]. To target the multifacets of AD, we designed novel pyrrolo[2,3-*b*]pyridinyl-based GSK-3β inhibitors incorporating 8-hydroxyquinoline motif to also target AD metal dyshomeostasis. The synthesis of B10 is shown in Figure 1.

5-Bromo-3-iodo-1-(phenylsulfonyl)-1*H*-pyrrolo[2,3-*b*]pyridine (1) was easily prepared by the reaction of 5-bromo-3-iodo-1*H*-pyrrolo[2,3-*b*]pyridine with benzenesulfonyl chloride according to reported methods [46]. Suzuki coupling of 1 with 8-methoxy-6-(4,4,5,5-tetramethyl-1,3,2-dioxaborolan-2-yl)quinoline [47] catalyzed by Pd(dppf)Cl_2_, followed by the removal of benzenesulfonyl protective group under basic conditions and the subsequent removal of the ether methyl group by hydrobromic acid, generated the 8-hydroxyquinoline derivative 2 in 47.8% yield (3 steps). The reaction of 2 with *tert*-butyldimethylsilyl chloride (TBSCl) in DMF in the presence of imidazole gave the TBS ether 3 in 60% yield. The Suzuki coupling of 3 with *N*-(4-(4,4,5,5-tetramethyl-1,3,2-dioxaborolan-2-yl)benzyl)pyridin-4-amine catalyzed by Pd(PPh_3_)_4_ in the presence of Na_2_CO_3_ in ethanol and water at 100 °C, followed by the removal of the TBS protective group with tetra-*n*-butylammonium Fluoride (TBAF), furnished B10 in 32% yield.

### 3.2. B10 Is a Potent GSK-3β In Vitro Inhibitor

**Figure 2:**
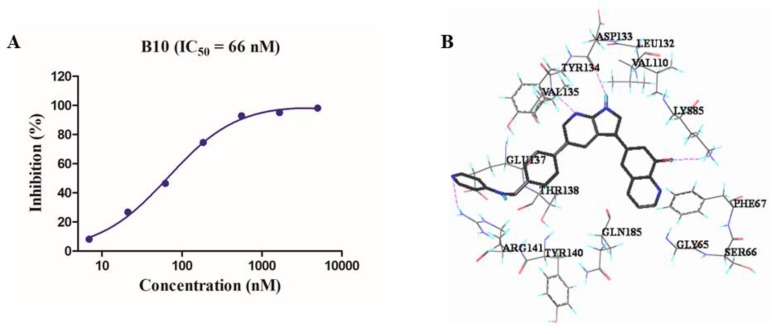
(**A**) The inhibition curve of B10 against GSK-3β; (**B**) the predicted binding mode of B10 in the ATP binding pocket of GSK-3β (PDB ID: 5F95).

Staurosporine is a prototypical ATP-competitive kinase inhibitor that potently inhibits GSK-3β with a reported IC_50_ value of 15 nM [48]. In the caliper mobility shift assay, staurosporine was used as a positive control with a determined IC_50_ of 16.5 ± 1.2 nM (n = 3). B10 was found to be a potent GSK-3β inhibitor with an IC_50_ of 66 ± 2.5 nM (Figure 2A).

To explore the possible interaction mode of the B10 with GSK-3β, molecular docking studies were performed on SYBYL based on the reported GSK-3β structure (PDB ID: 5F95) [49]. As shown in Figure 2B, B10 fitted well into the ATP binding pocket of GSK-3β: the pyrrolo[2,3-*b*]pyridinyl moiety formed two hydrogen bonds with VAL135 and ASP133 in the hinge region; the 8-hydroxyquinoline scaffold occupied one hydrophobic pocket and made crucial hydrogen bonding with LYS85 through its 8-hydroxyl group; the pyridin-4-amine portion fell into another hydrophobic pocket and formed hydrogen bond interaction with ARG141 through the pyridine nitrogen atom. All these hydrophobic interactions and hydrogen bonds may contribute to its high potency against GSK-3β.

### 3.3. B10 Regulates GSK-3β and β-catenin Signal Pathways and Inhibits Tau Phosphorylation in Human Neuroblastoma SH-SY5Y Cells

**Figure 3:**
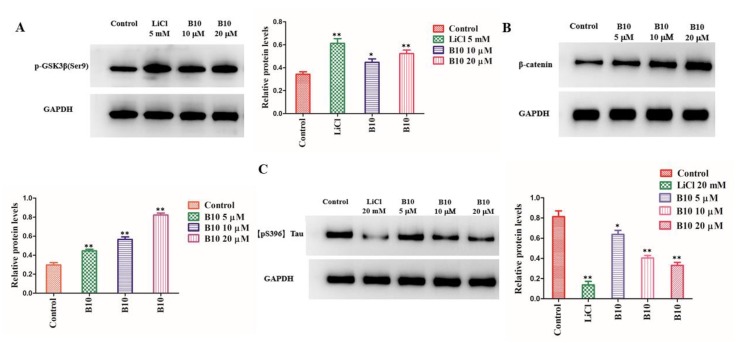
B10 is a potent GSK-3β inhibitor in SH-SY5Y cells. (**A**) The effect of B10 on phosphorylation of GSK-3β at the site of Ser9; (**B**) the effect of B10 on β-catenin abundance; (**C**) the effect of B10 on phosphorylation of tau at Ser396. Protein expression was detected by immunoblot analysis with a specific antibody. Values represent the mean ± SD (n = 3); * *p* < 0.05, ** *p* < 0.01 vs. control.

Since B10 showed high potency in the inhibition of GSK-3β at the enzymatic level, we further investigated its effect on SH-SY5Y cells by Western blot assays. LiCl is a non-ATP competitive inhibitor of GSK-3β. At the concentration of 5 mM, LiCl greatly increased the levels of GSK-3β phosphorylated at Ser9 (p-GSK-3β/GAPDH: 0.62 ± 0.057 vs. 0.35 ± 0.012), an inactive form of GSK-3β (Figure 3A). Treatment with B10 at 10 μM and 20 μM dose-dependently increased the phospho-GSK-3β level in comparison with the control group, and the p-GSK-3β/GAPDH ratio was 0.44 ± 0.037 and 0.53 ± 0.045, respectively.

β-Catenin is a Wnt signaling component that is destabilized upon that activation of GSK-3β [50]. In agreement with its inhibitory activity of GSK-3β on SH-SY5Y cells, B10 increased β-catenin abundance in a dose-dependent manner (Figure 3B). After the treatment with B10 at the concentrations of 5 μM, 10 μM, and 20 μM, the β-catenin/GAPDH ratio increased from 0.30 ± 0.016 of the control to 0.46 ± 0.033, 0.57 ± 0.051, and 0.83 ± 0.086, respectively.

Elevated levels of hyperphosphorylated tau are highly related to the formation of NFTs in the brains of AD patients. When SH-SY5Y cells were treated with 20 µM Aβ_25–35_ for 6 h, the phosphorylation of tau protein at Ser396 increased significantly with a p-tau/GAPDH ratio 0.83 ± 0.061 (Figure 3C). Treatment with B10 at 5 μM, 10 μM, and 20 μM resulted in a decrease in tau phosphorylation level in a concentration-dependent way (p-tau/GAPDH: 0.65 ± 0.029, 0.40 ± 0.061, and 0.33 ± 0.065). At the concentration of 20 µM, more than half of the hyperphosphorylated tau was reduced by pretreatment with B10.

### 3.4. B10 Selectively Chelates with Fe^2+^, Zn^2+^, Cu^2+^, and Al^3+^

**Figure 4:**
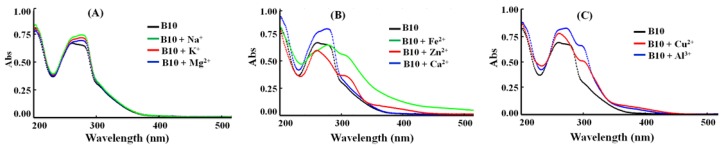
UV-vis spectra of B10 (20 μM) alone or in the presence of metal ions (40 μM) in CH_3_OH. (**A**) Na^+^, K^+^, Mg^2+^; (**B**) Fe^2+^, Zn^2+^, Ca^2+^; (**C**) Cu^2+^, Al^3+^.

Compound B10 was evaluated for its chelating ability with Na^+^, K^+^, Mg^2+^, Fe^2+^, Zn^2+^, Ca^2+^, Cu^2+^ and Al^3+^ by the UV−vis spectroscopy assay [51]. When Na^+^, K^+^, Mg^2+^, or Ca^2+^ was mixed with B10, the electronic spectra showed no obvious changes (Figure 4A,B), indicating B10 has little chelating ability with these metal ions. When FeSO_4_ was mixed with B10, a red shift from 262 nm to 280 nm were observed, and a new peak appeared at 310 nm (Figure 4B). In the presence of ZnCl_2_, CuCl_2_, or AlCl_3_, new absorptions at 311, 304, and 302 nm were observed, respectively. These results indicated that B10 could chelate effectively with these ions. There is a controversy over the role of Al^3+^, increasing evidence supports the implication of it in the development of AD [52]. The selective chelating ability of B10 toward Fe^2+^, Zn^2+^, Cu^2+^ and Al^3+^ makes it as a potential agent targeting metal dyshomeostasis in AD.

### 3.5. B10 Has a Direct Effect on Aβ_1−42_ Aggregation and Disaggregation of Aβ Aggregates and Affects Cu^2+^-Induced Aβ_1−42_ Aggregation and Cu^2+^-Aβ_1−42_ Aggregates Disaggregation

**Figure 5:**
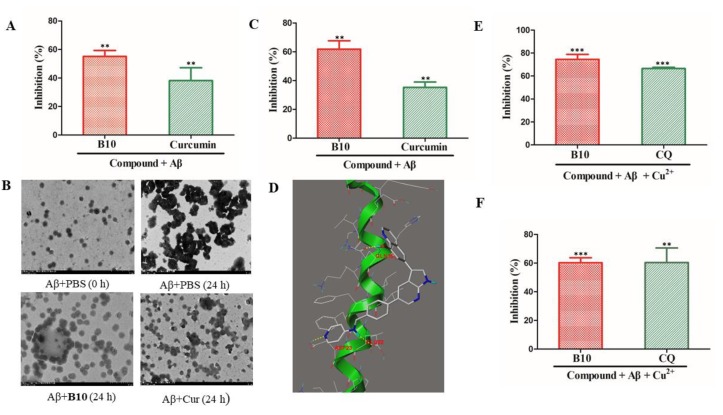
(**A**) Effect of B10 on the monomeric Aβ_1−42_ aggregation; (**B**) TEM images of Aβ_1−42_ aggregation; (**C**) effect of B10 on Aβ_1−42_ aggregates disaggregation; (**D**) molecular docking of B10 with Aβ_1−42_ (PDB code: 1IYT); (**E**) effect of B10 on Cu^2+^-induced Aβ_1−42_ aggregation; (**F**) effect of B10 on Cu^2+^-Aβ_1−42_ aggregates disaggregation. In aggregation assay, Aβ_1−42_/compound (1/1) or Aβ_1−42_/Cu^2+^/compound (1/1/2) was in HEPES at 37 °C for 24 h. In disaggregation assay, after Aβ_1−42_ or Aβ_1−42_/Cu^2+^ was incubated for 24 h to form aggregates, compounds were then added and further incubated for 24 h. Values are reported as the mean ± SD of three independent experiments. *** *p* < 0.001, ** *p* < 0.01 vs. Aβ_1−42_ or Aβ_1−42_/Cu^2+^ alone.

To determine whether B10 has a direct effect on the inhibition the aggregation of Aβ monomers, we incubated monomeric Aβ_1−42_ (20 μL, 40 µM) with B10 (20 μL, 40 µM) and quantified the amount of Aβ aggregates by thioflavin T (ThT) fluorescence assay with curcumin (cur) as a positive control [53]. As shown in Figure 5A, B10 effectively blocked the Aβ aggregate formation with an inhibitory rate of 55.1 ± 3.0%, more potent than curcumin (38.1 ± 6.3%). This was confirmed by TEM images (Figure 5B). In addition, when B10 was incubated with solutions containing preformed Aβ aggregates, B10 effectively disaggregated the Aβ fibrils, reducing the amount of Aβ aggregates by 61.8 ± 4.1%, much more potent than curcumin (35.3 ± 2.6%) (Figure 5C).

To elucidate why B10 affects Aβ_1−42_ aggregation and disaggregation, docking simulations were carried out based on the resolved structure of the peptide in its α helix form (PDB code: 1IYT). In Aβ_1−42_ monomer, the self-recognition hydrophobic core “^16^KLVFFA^21^” is located in the central region of the Aβ peptide and is known as the key amyloidogenic sequence that initiates the Aβ−Aβ interaction [54]. As shown in Figure 5D, B10 interacted with the “^16^KLVFFA^21^” amyloid region. The pyridyl nitrogen atom formed a hydrogen bond with Asp23, and the pyridine ring interacted with Phe20 through π−π stacking. The 4-pyridylamino NH served as a hydrogen bond donor to interact with Glu22. The hydroxyl group in the 8-hydroxyquinoline moiety formed a hydrogen bond with Gln15. All of these interactions may contribute to the direct effect of B10 on Aβ_1−42_ aggregation and disaggregation.

Cu^2+^ is a well-known metal ion that modulates the Aβ aggregation and toxicity [42]. To validate whether B10 could target metal dyshomeostasis, the mixtures of Aβ_1−42_ (20 μL, 40 µM), CuCl_2_ (20 μL, 40 µM), and B10 or CQ (40 μL, 40 µM) were incubated at 37 °C for 24 h and detected by ThT assay. In the presence of Cu^2+^, the fluorescence intensity of the Aβ_1−42_ and Cu^2+^ mixture significantly increased over 1.2-fold in comparison with that of Aβ_1−42_ alone. As shown in Figure 5E, the positive control CQ, a known nonspecific copper/zinc chelator that can reduce Aβ deposits and improve learning and memory capacities of APP transgenic mice [41], inhibited the Cu^2+^-induced Aβ_1−42_ aggregation by 66.5 ± 0.9%. B10 was more potent than CQ with an inhibitory rate of 74.6 ± 3.5%.

As an effective agent targeting AD copper homeostasis, the chelator is required not only to inhibit Cu^2+^-induced Aβ aggregation but also to have the ability to extract the copper ion from the aggregates and promote their disaggregation. To evaluate the activity of B10 to disaggregate Cu^2+^-induced Aβ_1−42_ aggregation, Aβ_1−42_ and Cu^2+^ were first incubated at 37 °C for 24 h to form Aβ_1−42_ fibrils, B10 or CQ was then added and incubated for another 24 h. As shown in Figure 5F, B10 showed a similar effect as that of CQ, reducing the Cu^2+^-Aβ_1−42_ oligomers by 60.3 ± 3.5%.

### 3.6. B10 Promotes Neuronal Neurite Outgrowth and Growth-Associated Protein 43 (GAP43), N-myc, and Microtubule-Associated Protein 2 (MAP-2) Expressions in SH-SY5Y Cells

**Figure 6:**
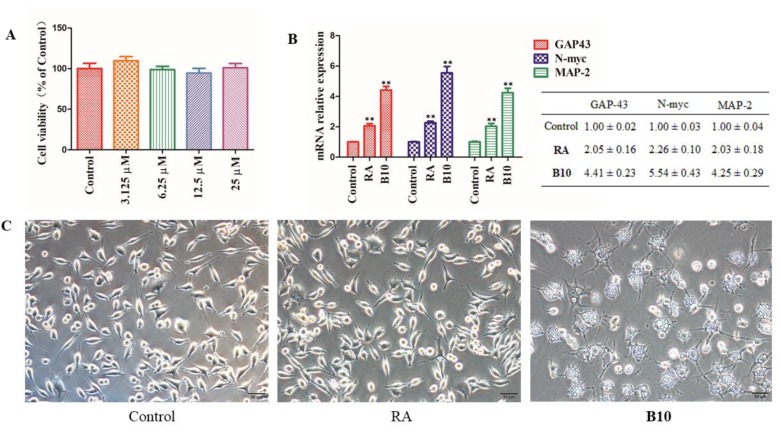
(**A**) Effects of B10 on SHSY5Y cell viability, *p* > 0.05 (n = 3); (**B**) effect of B10 or RA (10 μM) on neurogenesis markers expression (GAP43, N-myc and MAP-2) in SHSY5Y cells (24 h), ** *p* < 0.01 vs. control, n = 3; (**C**) effects of 1 B10 or RA (10 μM) on neurite outgrowth (72 h). Pictures were taken at 200× magnification.

Since differentiated SH-SY5Y cells possess similar morphology and biochemical processes to mature neurons, they were widely used to study neuronal activity [55]. In cell viability assay, B10 showed no cytotoxicity towards SH-SY5Y cells up to 25 μM (Figure 6A). B10 was further evaluated for its ability to induce neurogenesis in SH-SY5Y cells.

There are several recognized biomarkers for neurogenesis, and among them, the growth-associated protein 43 (GAP-43) plays a role at synaptic level [56] and the N-myc gene is essential for normal neurogenesis and regulates cell proliferation, differentiation, and nuclear size [57], while the microtubule-associated protein 2 (MAP-2) is abundant in the mammalian nervous system and associated with the neurites and dendrite scaffold formation [58]. RA is a vitamin A metabolite that is widely used in the differentiation of SH-SY5Y cells into highly homogeneous populations of neuron-like cells [59]. After the treatment of SH-SY5Y cells with B10 or RA (10 μM) for 24 h, the mRNA expression of GAP43, N-myc, and MAP-2 was assessed by RT-PCR analysis. As shown in Figure 6B, RA induced significant expression of all the three markers. In comparison with RA, B10 exhibited more profound effects and was more than 2-fold more active than RA in promotion of the expression of GAP43, N-myc, and MAP-2 in SH-SY5Y cells. To confirm the obtained results, the morphology of the differentiated neuronal neurite outgrowth was assessed after cell treatment for 72 h with B10 or RA (10 μM). In agreement with the RT-PCR results, B10 induced more significant neurite outgrowth than RA (Figure 6C).

## 4. Discussion

Due to the complexity of AD pathogenesis, there is no effective treatment to prevent or reverse the progression of AD, and the discovery of effective anti-AD drugs is rather challenging. In recent years, the design of a multifunctional inhibitor that target multifacet of AD was a well-accepted strategy. In this study, we designed a novel GSK-3β inhibitor B10 as a multitarget-directed ligand through incorporating the 8-hydroxyquinoline motif in the molecule to target AD metal dyshomeostasis.

GSK-3β is an important target for the treatment of AD because it acts as a linker between the two important histopathological hallmarks: NFTs formed by the accumulation of hyper-phosphorylated tau protein, and the extracellular senile plaques caused by abnormal Aβ aggregation. The overactivation of GSK-3β is highly related to the abnormal tau hyperphosphorylation that plays a crucial role in axonal assembly associated with synaptic transmission, neuronal degeneration and dysfunction, and NFTs. B10 was proven to be a potent GSK-3β inhibitor. It effectively increased the inactivated form of GSK-3β through the phosphorylation at Ser9 and reduced the phosphorylation of tau protein at Ser396 in SH-SY5Y cells.

Aβ protein, the main component of senile plaques, is a hydrophobic, intrinsically disordered peptide with 36−43 amino acids [60]. Aβ is generated by the sequential metabolism of amyloid precursor protein by β- and γ-secretases, and Aβ_1−42_ is the most toxic and predominant species found in plaques [61]. Soluble monomeric Aβ_1−42_ is prone to self-assembly to form Aβ oligomers that aggregate to form protofibrils and then mature amyloid fibrils. There is increasing evidence that the soluble prefibrillar Aβ oligomers are more toxic than mature fibrils [62]. Therefore, an ideal anti-AD drug should have the ability to prevent the formation as well as to disaggregate the Aβ oligomers and fibrils. Though GSK-3β has some effect on Aβ formation through its regulation of γ-secretase [32], the inhibition of GSK-3β may have limited effects on reducing the Aβ level, considering the complicated processes in Aβ formation and its clearance. In this regard, B10 is unique as a potent GSK-3β inhibitor that can inhibit Aβ oligomers formation and promote Aβ oligomers and fibrils disaggregation. As far as we know, B10 is the first potent GSK-3β inhibitor reported to play a direct role in Aβ. Molecular docking simulations revealed the binding mode of B10 with Aβ_1−42_ in the self-recognition hydrophobic “^16^KLVFFA^21^” motif.

The homeostasis dysregulation of transition metal ions (Cu^2+^, Zn^2+,^ and Fe^2+^), especially Cu^2+^, not only promotes the aggregation and deposition of Aβ, but also generates ROS, leading to oxidative stress [41,42]. Metal chelating agents, such as CQ, can prevent or reverse copper-Aβ interactions and reduce plaque burden in the brain of transgenic AD mouse models with marked improvements in cognitive performance [63,64]. Clinical investigations proved that CQ or its derivative could improve cognitive scores and reduce Aβ levels in AD patients [65,66]. These studies suggest that targeting copper dyshomeostasis provides a choice for AD therapy. By introducing an 8-hydroxyquinoline moiety as a bidentate chelator, B10 selectively chelates with AD-related metal ions, inhibits Cu^2+^-induced Aβ_1−42_ aggregation, and promotes the disaggregation of Cu^2+^- Aβ_1−42_ oligomers, serving as a potent ligand to target AD metal dyshomeostasis.

A recent study confirms that new neurons are born throughout aging in healthy humans, but this drops sharply in AD patients, suggesting that therapeutic strategies aimed at increasing neurogenesis may slow the disease [67]. β-Catenin is a substrate of GSK-3β. Phosphorylation of β-catenin in the absence of Wnt signaling by GSK3β results in its ubiquitination and subsequent degradation by proteasomes [68]. As a necessary transcriptional coactivator, β-catenin not only influences cellular events but also plays important roles in cell adhesion complexes, including those necessary for neuronal differentiation. The depletion of β-catenin by small interfering RNA blocked the neurite outgrowth in SH-SY5Y cells [69]. As a potent GSK-3β inhibitor, B10 effectively increases the β-catenin abundance, promotes neuronal neurite outgrowth, and induces significant expression of GAP43, N-myc, and MAP-2 in SH-SY5Y cells. These results are probably a combined effect of B10 on the GSK-3β and β-catenin signal pathways.

## 5. Conclusions

Based on the GSK-3β crystal structure and through rational drug design and molecule docking simulations, we designed and screened novel pyrrolo[2,3-*b*]pyridine-based GSK-3β inhibitors with the incorporation of 8-hydroxyquinoline motif as a bidentate ligand to target AD metal dyshomeostasis as well. Compound B10 was identified as a potent GSK-3β inhibitor. Unlike other GSK-3β inhibitors reported, B10 is unique and has a direct effect on Aβ, inhibiting the Aβ oligomers formation and promoting Aβ oligomers and fibrils disaggregation. B10 also targets tau and metal dyshomeostasis. Moreover, B10 is more potent than RA in promoting neuronal neurite outgrowth and in inducing GAP43, N-myc, and MAP-2 expressions in SH-SY5Y cells, possibly through the GSK-3β and β-catenin signal pathways. Therefore, B10 could serve as a good lead for future structural optimization and further in vivo investigations as a multifunctional agent for the treatment of AD.
